# Adoption of an Electronic Decision Support Tool for Capacity Building of Community Health Workers: Mixed Methods Study

**DOI:** 10.2196/69874

**Published:** 2026-01-07

**Authors:** Anton Elepaño, Carol Stephanie Tan-Lim, Clare Bankhead, Leonila Dans, Noleen Marie Fabian, Josephine Sanchez, Antonio Dans, Catherine Pope

**Affiliations:** 1 Nuffield Department of Primary Care Health Sciences Medical Sciences Division University of Oxford Oxford United Kingdom; 2 National Telehealth Center National Institutes of Health University of the Philippines Manila Manila, National Capital Region Philippines; 3 Center for Integrative and Development Studies University of the Philippines Diliman Quezon City Philippines; 4 Department of Clinical Epidemiology College of Medicine University of the Philippines Manila Manila Philippines; 5 Health Equity and Research Foundation St. Luke's Medical Center Taguig Philippines

**Keywords:** community health workers, clinical decision support, health systems research, digital health, UpToDate, quantitative analysis

## Abstract

**Background:**

Complimentary subscriptions to UpToDate, a decision support tool, were provided to community health workers (CHWs) in rural and remote primary care sites as part of a government-funded health system research program. A feasibility evaluation conducted after the first year of implementation showed that UpToDate was acceptable among CHWs despite infrastructural barriers.

**Objective:**

This follow-up study evaluated the longitudinal adoption of UpToDate among CHWs and examined how sociocultural, political, and environmental factors influenced its use. Drawing on the nonadoption, abandonment, scale-up, spread, and sustainability framework, this study aimed to understand not only use patterns but also broader challenges to scale-up, spread, and sustainability in a complex health system.

**Methods:**

An explanatory mixed methods design was used combining analysis of use and program activity logs; program reports; and focus groups with CHWs, health care professionals, and program implementers. Quantitative analysis of use logs (March 2021 to September 2023) compared adoption over time by using descriptive statistics, CIs, and chi-square tests. Qualitative data came from the reanalysis of previous focus group transcripts and program reports and from a new focus group with program implementers. Reflexive thematic analysis was used to interpret how CHWs and implementers perceived and used the tool, and findings were integrated to explain quantitative trends.

**Results:**

Use of UpToDate was modest and declined over time. Monthly active use among CHWs and midwives fell substantially from 3.57% (97/2720 person-months) in 2021 to 1.07% (37/3456) in 2022 and remained low at 1.50% (39/2592) up to 2023, with markedly higher engagement in the rural site than in the remote site. Peaks in use coincided with program activities, whereas prolonged troughs followed typhoons, power outages, and other disruptions. Log data showed that users primarily consulted patient education articles rather than clinician-oriented decision tools. Qualitative analyses revealed that CHWs appropriated UpToDate as a learning aid and source of professional credibility. Structural shocks, heavy workloads, language barriers, and limited device access constrained individual use, and communal practices (shared devices and learning activities) meant that meaningful engagement often went unrecorded in vendor metrics.

**Conclusions:**

Our findings show that acceptability does not guarantee sustained use and that adoption cannot be captured fully by log-in counts. UpToDate’s value for CHWs lay in how it was domesticated as a tool for building capacity and professional credibility, not in its intended function as a decision aid used at the point of care. Therefore, evaluations of digital health tools should incorporate indicators of learning and social capital alongside use metrics. Policymakers should recognize that infrastructural fragility and communal adaptation shape digital health uptake. Embedding tools into ongoing training and peer learning structures, providing offline and multilingual support, and investing in resilience planning will be crucial for meaningful scale-up and sustainability.

## Introduction

Community health workers (CHWs) are locally selected health aides who live in the communities they serve, are accountable to those communities, receive support from the health system without being fully integrated into it, and generally undergo less training than professional health workers [1]. Their responsibilities include delivering health education, providing basic care, and referring patients to formal services. However, their ability to perform these roles is constrained by limited access to reliable health information and ongoing training [2,3]. Digital health interventions (DHIs) offer potential solutions to these capacity gaps. Tools such as mobile apps, clinical guideline repositories, and point-of-care decision support systems have been deployed to support CHWs, but most are limited to pilot projects or narrowly scoped disease programs [4-7].

UpToDate is a commercial point-of-care tool [8] and web-based information resource [9] that covers a broad range of health topics and decision tools. Systematic evaluations among point-of-care summaries have shown that no single tool consistently outperforms across all domains; for example, DynaMed and eMedicine scored highest for volume of coverage, whereas Clinical Evidence and UpToDate tied for editorial quality [10]. A later independent review similarly found that, while several tools met high quality standards, DynaMed and UpToDate were among those consistently scoring high in terms of breadth and editorial rigor, whereas others such as Medscape were less consistent in transparency and referencing [11].

In this study, UpToDate was chosen for implementation not only for its broad content and usability but also for its inclusion of a structured patient education corpus (“The Basics” and “Beyond the Basics”) written in lay language. Taken together, these factors justified its selection as one of the interventions in the Philippine Primary Care Studies (PPCS). The PPCS is a government-funded health system research program supporting universal health care reforms. From 2019 to 2023, the PPCS program provided free UpToDate subscriptions to CHWs and other frontline workers in rural and remote primary care sites accompanied by onboarding sessions and journal club sessions (ie, small group meetings where participants discuss clinical cases and related evidence).

In 2021, a feasibility evaluation drawing on the technology acceptance model (TAM) framework was conducted after the first year of implementation of the free UpToDate subscriptions [12]. This evaluation identified barriers to the uptake of UpToDate, which included digital literacy, limited language options, and poor information and communications technology infrastructure [13]. Despite these barriers, the technology was highly acceptable among CHWs, a notable finding given that the features were not specifically designed for this cadre.

TAM was appropriate for that feasibility phase as it is widely validated to predict technology adoption, including in resource-limited settings [14]. However, while TAM can capture acceptability and intention to use, its focus on individual and organizational acceptance means that it is less able to interrogate the infrastructural and political factors that shape sustained use [15]. Moreover, previous evaluations using TAM have not reviewed vendor longer-term use data, leaving a gap on whether expressed acceptability translated into continued adoption. To address this, this study also used the nonadoption, abandonment, scale-up, spread, and sustainability (NASSS) framework [16,17], which was developed to explain why digital health technologies are variably adopted or abandoned and how complexity across multiple domains shapes long-term outcomes. The NASSS framework comprises 7 interacting domains: the condition, the technology, the value proposition, the adopter system, the organization, the wider system, and adaptation over time. It has been applied to complex health technologies, including video-based outpatient consultations, remote monitoring systems for chronic illness, and assistive technologies for older adults [17].

This study addressed the research gap by providing the first formal analysis of adoption patterns and examining broader dimensions of value beyond vendor-defined metrics, situated within broader infrastructural, political, and system-level challenges across NASSS domains. Specifically, this study was guided by two primary research questions: (1) How does UpToDate fit into the CHWs’ roles in primary care? (2) How do the wider sociocultural, political, and environmental systems influence the adoption of UpToDate among CHWs?

## Methods

This manuscript followed the Checklist for Mixed Methods Research Manuscript Preparation and Review [18] (Multimedia Appendix 1) and the Standards for Reporting Qualitative Research for the qualitative components [19] (Multimedia Appendix 2).

### Setting

Within the PPCS program sites, only those with CHWs were selected. These included 2 sites: Bulusan, a geographically isolated municipality bordered by the Pacific Ocean (hereafter referred to as the “remote site”) and the municipality of Samal, situated west of Manila Bay (the “rural site”). Both sites exhibited a health care professional density of 0.01 per 10,000 people [20], starkly below the estimated requirement of 25 per 10,000 for adequate primary care service coverage [21].

### Study Design

**Figure 1:**
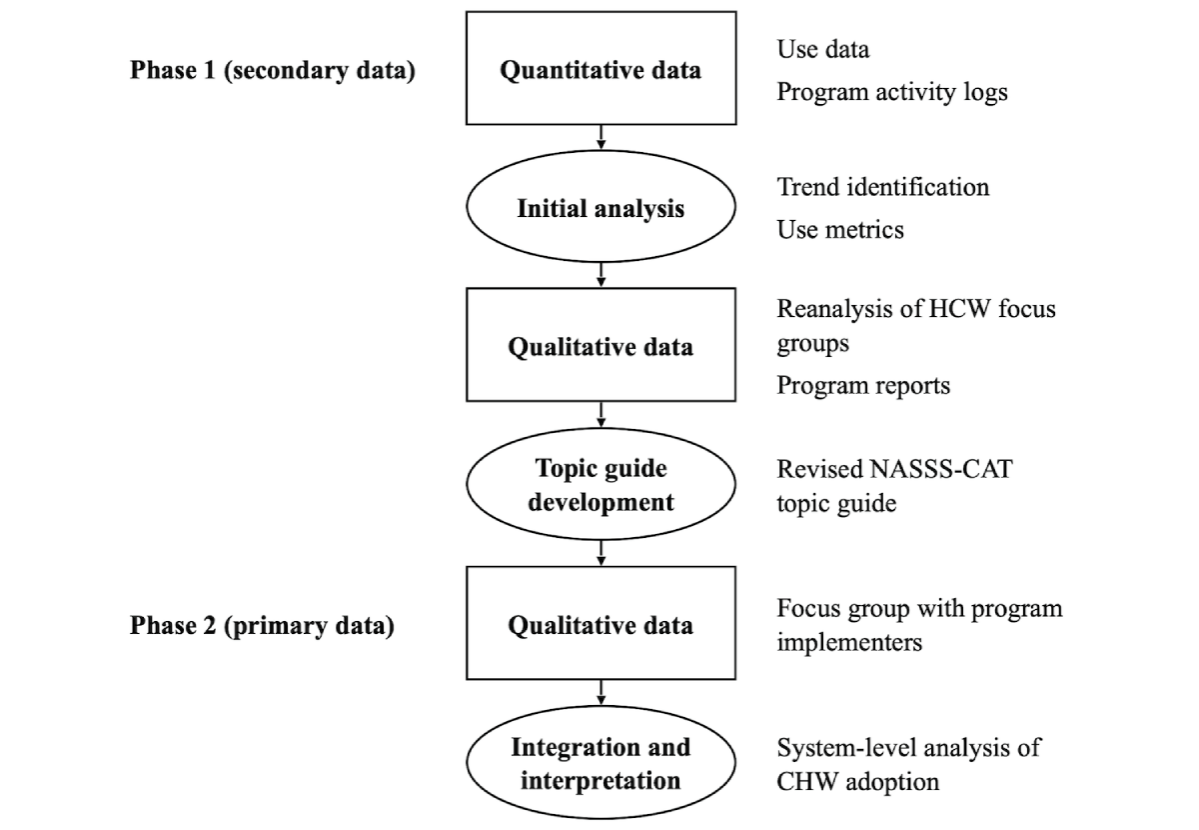
Multiphase explanatory mixed methods study flowchart. CHW: community health worker; HCW: health care worker; NASSS-CAT: nonadoption, abandonment, scale-up, spread, and sustainability framework complexity assessment tool.

This study used a multiphase explanatory mixed methods approach (Figure 1). Quantitative analysis of vendor use data first provided a population-level description of adoption patterns among CHWs and midwives. These findings then informed a rereading of existing program reports and transcripts of focus group with health workers. Because use data had not been available in previous evaluations, this rereading allowed previous findings on acceptability to be revisited through a new lens, highlighting inconsistencies between reported intention to use and actual adoption. Together, the quantitative findings and this secondary qualitative analysis pointed to missing perspectives, particularly regarding organizational processes and wider system influences. To address these gaps, we developed a targeted interview guide (Multimedia Appendix 3) for program implementers, who were best positioned to comment on these broader domains. Integration took place after analysis of quantitative and qualitative data, where use patterns, frontline accounts, and implementer perspectives were brought together to produce a more comprehensive interpretation of adoption and nonadoption.

This sequencing allowed the researchers to zoom out to identify population-level trends, zoom in to explore individual and organizational experiences, and zoom out again to situate these micro-level insights within broader system dynamics. This layered approach provided a more comprehensive analysis and minimized bias from relying solely on decontextualized metrics or from overly narrow qualitative sampling.

### Reflexivity and Positionality

This study was conducted within an interpretivist paradigm, which recognizes multiple participant meanings regarding the adoption and nonadoption of UpToDate. Purposive sampling was used to capture a range of perspectives from users and program implementers, and reflexive thematic analysis was used to interpret the data [22]. The research team comprised clinicians and digital health researchers, some with previous involvement in the PPCS program and related health system research. Collectively, the team drew on experiences with underserved communities, which informed a sensitivity to power and equity issues. This positionality facilitated access and contextual understanding but also carried risks of interpretive bias, which were mitigated through team-based discussion and iterative cross-checking against the raw data.

### Phase 1: Secondary Data Collection and Analysis

#### Quantitative Data

Use data from 2021 to 2023 stratified by health care provider type were requested from UpToDate’s service provider, Wolters Kluwer. These data were available only in monthly intervals and in aggregated form, precluding matching specific users with their use frequency. Physicians and nurses were reported as separate categories, whereas CHWs and midwives were combined by the vendor into an “Others (CHW/midwife)” category, reflecting the novelty of the technology for these user types. As a result, our quantitative analysis focused on this aggregated subgroup of CHWs and midwives. All CHWs and midwives enrolled in the PPCS program and who were provided with access to UpToDate between 2021 and 2023 were included in the vendor use data. Subscriber counts were based on annual employee data of health care workers assigned to each barangay (the smallest administrative unit in the Philippines). For analysis, we treated each subscribed CHW or midwife as contributing one subscriber person-month for each calendar month in which their account was active.

As a measure of adoption, monthly active users (MAUs) were defined as the proportion of registered CHW and midwife subscribers who logged at least one session in that month; the denominator was the number of subscribed CHWs and midwives at the site that month. From March 2021 to September 2023, CHWs and midwives comprised 89.04% (7807/8768 person-months) and 10.96% (961/8768) of the subscribers, respectively (remote site: 4481/4760, 94.14% CHWs; rural site: 3326/4008, 82.98% CHWs). Estimated MAUs are presented with Wilson 95% CIs. Sessions per user were defined as total sessions divided by the number of unique active users in that month and were summarized monthly.

For inferential statistics, observations were aggregated to nonoverlapping calendar years within the window (2021 to 2023). We first conducted chi-square tests of homogeneity on aggregated counts: a 3 × 2 test comparing years pooled across sites and a 2 × 2 test comparing sites across the window. To quantify effects, we reported absolute percentage point differences with 95% CIs computed using the Newcombe score method for independent proportions (year to year within each site, year to year with both sites pooled, and the overall contrast between the rural and remote sites).

Content type measures the number of user interactions for each topic category defined by Wolters Kluwer. This content is segmented vertically by specialties (eg, dermatology, gastroenterology, and psychiatry), horizontally by levels of care (eg, primary care and patient education), and by app features (eg, calculators and clinical pathways). This metric was assessed as a percentage of total content views per month to allow for inferences about different uses of UpToDate.

Program activity logs were obtained from the program secretariat, who supplied records of CHW-led journal club sessions supervised by the PPCS. These dates were superimposed on plots of MAUs and sessions per user to infer associations between use and the engagement of program implementers with users.

#### Qualitative Data

Data from 2 sets of focus groups were reanalyzed in this phase. The first was conducted by Calderon et al [13] in April 2021 to assess the feasibility and acceptability of UpToDate. The second was conducted by De Mesa et al [23] in July 2023 to explore barriers to role performance among CHWs. Eligible participants in the study by Calderon et al [13] were all UpToDate subscribers (CHWs, physicians, midwives, and nurses), whereas the study by De Mesa et al [23] included only CHWs. Both studies used a pilot-tested semistructured topic guide adapted from a similar study [24], which elicited discussions regarding the feasibility and acceptability of using UpToDate in relation to health care worker roles. The study by Calderon et al [13] included 4 online focus groups of 4 to 8 participants each, whereas the study by De Mesa et al [23] included 6 in-person focus groups of 5 to 7 participants each. Sessions were moderated in Bikol, English, and Filipino languages by trained multilingual research assistants. Audio recordings were manually transcribed, anonymized, and translated into English.

A reanalysis of focus group transcripts from the studies by Calderon et al [13] and De Mesa et al [23] was undertaken to address questions not covered in the original studies. Although the focus groups were designed to explore different research questions, the breadth of the discussions provided sufficient material relevant to adoption, particularly within the adopter and technology domains of the NASSS framework. Program reports comprising published and unpublished PPCS manuscripts were assessed for relevance to the research questions. Relevant reports provided social context and organizational descriptions of the remote and rural settings.

Following the reflexive thematic analysis approach by Braun and Clarke [22,25], coding of transcripts and program reports was undertaken (by AE) as an interpretive process to identify meaningful patterns in the data. Initial codes were developed by reading through the interviews. Further reading and coding were informed by concepts and domains from the NASSS framework. Codes were organized and managed using NVivo (version 14; Lumivero). To support theme development, a “one sheet of paper” method was used to visually map how codes clustered and related to one another. Candidate themes were then developed through iterative reflection and sensemaking, and team discussion explored coherence and distinction in these themes. Themes were reviewed and further refined by returning to the full dataset to ensure that they meaningfully represented the complexities of participant accounts. Preliminary themes were integrated with quantitative findings to explain use patterns and identify knowledge gaps, particularly about the wider system, where health care worker perspectives were sparse. While this phase primarily focused on the technology, adopter, and condition domains of the NASSS framework (consistent with the a priori TAM lens), it offered limited coverage of the remaining NASSS constructs. These gaps informed the development of a topic guide (Multimedia Appendix 3) used for the phase 2 focus group based on the NASSS complexity assessment tool [26].

### Phase 2: Primary Data Collection and Analysis

This phase involved collecting qualitative data from an online focus group conducted in April 2024 with 6 PPCS program implementers involved in the UpToDate deployment. Participants were selected based on their involvement with PPCS in any capacity, including protocol development, data collection, data analysis, and manuscript writing. This phase addressed the second research question by providing social, organizational, and policy contexts not covered in detail in phase 1. The focus group with program implementers was moderated, audio recorded, manually transcribed, and anonymized by the lead author.

Transcripts were coded (by AE) using a theory-driven approach informed by the NASSS framework [17], enabling structured yet flexible analysis of how the intervention interacted with its evolving implementation context [16,27]. Coding procedures mirrored those used in phase 1. The NASSS domains guided analysis by mapping responses to relevant constructs. For example, organizational enablers were categorized under the organization domain, whereas system-level constraints were captured under the wider system domain. These codes were integrated with phase 1 data to refine internally coherent and analytically distinct themes [25] following the same reflexive and iterative approach described previously. To enrich the analysis, a coauthor not involved with the PPCS team (CP) also independently examined the qualitative data and contributed to discussions on coding and initial theme development (with AE).

### Integration and Interpretation

Candidate themes were reviewed (by AE and CP) and iteratively revised in relation to the entire dataset, encompassing findings from phases 1 and 2.

During this process, use metrics were re-examined alongside qualitative findings to explore associations between patterns of use and contextual factors such as user roles, implementation activities, and site-specific infrastructure. The content accessed on the UpToDate platform was interpreted in relation to how CHWs described using the tool and how this aligned with its intended functions as described by implementers and the service provider. Cross-site comparisons were also informed by available contextual data, such as internet connectivity and local implementation timelines. Triangulation was operationalized through this comparative process, integrating 3 sources of evidence: use analytics, CHW perspectives, and program implementer insights. Member checking of the final themes was conducted with program implementers to confirm the relevance and accuracy of interpretations.

### Ethical Considerations

Ethics approval was granted by the University of Oxford Medical Sciences Interdivisional Research Ethics Committee (R92309/RE001). This study served as an extension of the PPCS program and remained aligned with its original objectives. Ethical clearance for the broader program, including the previously collected qualitative data, was granted by the University of the Philippines Manila Research Ethics Board (2015-489-01) and the Department of Health Single Joint Research Ethics Board (2019-55). All participants provided informed consent, including consent for the future use of anonymized transcripts. Participants did not receive compensation for taking part in this study. All research data, including audio recordings and anonymized transcripts, were securely stored on encrypted Nexus365 university servers, with access restricted to the lead author. In accordance with ethics approvals, data will be retained for 3 years following publication and permanently deleted thereafter.

## Results

### Use Data and Program Activity Logs

**Figure 2:**
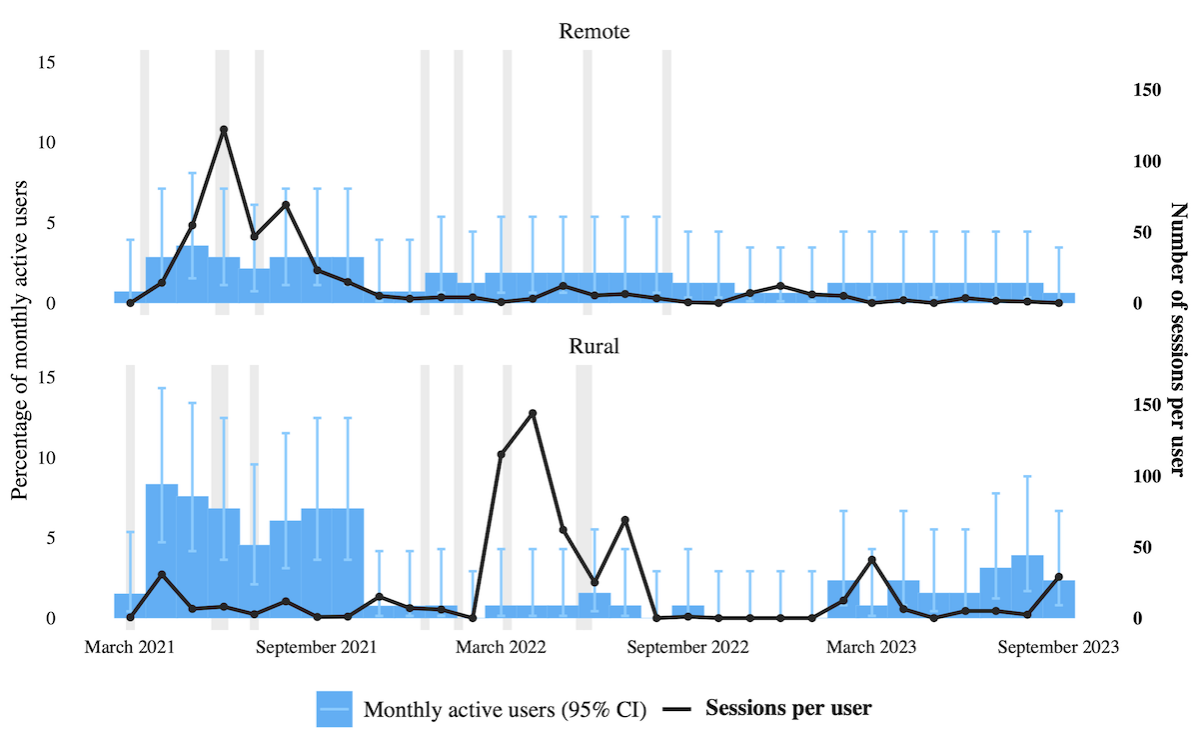
Use patterns of UpToDate among community health workers and midwives in the remote (Bulusan) and rural (Samal) sites across a 3-year period, with periods of engagement with the implementing agency highlighted in gray.

Use data spanning 31 months (March 2021 to September 2023) for the vendor-designated “other” user type (CHWs and midwives) were reviewed. Program activity logs from the implementing agency during the same period were also reviewed. The proportion of MAUs who were CHWs and midwives declined since the initial program evaluation in 2021 (Figure 2).

Adoption differed between the rural and remote sites between March 2021 and September 2023 (*P*=.006). CHW and midwife adoption in the rural site exceeded that in the remote site by 0.82 percentage points (95% CI 0.24-1.44). Pooled across sites, adoption differed across years (*P*<.001), and CHW and midwife monthly activity fell from 3.57% (97/2720 person-months) in 2021 to 1.07% (37/3456) in 2022 (−2.50 percentage points, 95% CI −3.32 to −1.75) and then did not clearly change in 2023 versus 2022 (+0.43 percentage points, 95% CI −0.13 to 1.06).

Engagement periods with program implementers coincided with higher MAU rates at both sites. The number of sessions per user varied and showed no clear association with the MAU rates or journal club sessions.

The service provider’s logs could not disaggregate CHW users from other health care professionals, highlighting the pragmatic challenges of tracking specific user types. Nevertheless, these data indicated that UpToDate was primarily used to search for patient education content (2424/6784 content views, 35.73% of total content views), a practice reinforced during CHWs’ onboarding and journal club sessions. This was followed by searching for infectious diseases (636/6784, 9.38%). Decision tools, including calculators (115/6784, 1.70%) and clinical pathways (5/6784, 0.07%) designed for medical professionals, represented only a minority of content views throughout the observation period.

### Integration and Interpretation

#### Focus Groups and Program Reports

Thematic analysis was undertaken using secondary data from 4 focus groups conducted by Calderon et al [13] and 6 focus groups conducted by De Mesa et al [23] and primary data from the additional focus group with program implementers. Most focus group participants (42/60, 70%) were CHWs, with all but one (41/42, 98%) being female (Table 1). Physician and nurse participants were generally younger than their CHW and midwife counterparts.

Five program reports that were relevant to the study’s research questions were included: 4 (80%) focused on the health workforce [13,23,28,29] and 1 (20%) focused on leadership and governance [30]. Through iterative rounds of coding and thematic analysis, two key themes were identified: (1) the multiplicity of intended uses and value propositions of the DHI and (2) acute shocks and everyday constraints to adoption.

**Table 1:** Characteristics of the focus group participants.

Participant type	Participants in the remote site, n	Participants in the rural site, n	Characteristics	
			Age (y), median (IQR)	Sex (female), n (%)
CHW^a^ (n=42)	23	19	46 (42-53)	41 (98)
Physician (n=4)	2	2	30 (26-46)	2 (50)
Midwife (n=4)	2	2	41 (33-53)	4 (100)
Nurse (n=4)	2	2	32 (30-42)	4 (100)
Program implementer^b^ (n=9)	4	5	46 (36-60)	3 (50)

^a^CHW: community health worker.

^b^Three program implementers were coordinators for both sites.

#### Theme 1: Multiplicity of Intended Uses and Value Propositions

One of the strongest patterns derived from the qualitative data was the plurality of intended uses of the intervention, which varied not only by design but also by stakeholder positioning. These uses included clinical decision support, educational programs (capacity building), and social capital.

##### Individual Clinical Decision Support (Technology)

For the vendor, UpToDate was marketed and envisioned primarily as a clinical decision support tool to be used by physicians at the point of care. Physicians echoed this, describing how the tool provided “summarized management guidelines at the tip of your finger” (D01), enabling them to manage patients locally rather than automatically referring them to higher-level facilities. This notion of decentralized care also filtered down to CHWs, who were increasingly positioned to support primary care through access to the same evidence base.

##### Part of an Educational Program (Organization)

However, for the implementers and program coordinators, the intervention’s perceived value proposition shifted toward capacity building. Implementers stressed how the tool was deployed less as a strict decision aid and more as a professional development and educational resource. One program coordinator noted the following:

Because CHWs are very well integrated in the community and trusted, they get asked about all sorts of conditions....UpToDate allows them to look for answers, even for rare diseases, if they are not familiar.

P06

This illustrates how implementers framed adoption in terms of capacity building, professional identity, and continuous training, positioning the technology within broader health system capacity agendas.

##### Symbolic and Social Capital (Adopters)

Among CHWs, UpToDate was reinterpreted less as a formal clinical tool and more as a form of symbolic and social capital. CHWs emphasized how access to the tool elevated their perceived authority (“UpToDate boosts my morale and confidence...” [C22]) and made them “trusted by clients for their knowledge” (P03). In contexts in which their legitimacy was fragile and their authority was frequently contested, having access to a globally recognized resource allowed them to anchor their advice in evidence, thereby enhancing credibility. In this way, UpToDate’s function exceeded its technical design; it became an instrument for reinforcing social standing and negotiating professional boundaries.

##### Quantitative Integration (Adoption Over Time)

This multiplicity of intended uses complicates how adoption trends are interpreted. Vendor-reported log-in data implicitly privilege the clinical decision support framing. As a result, alternative uses such as informal community teaching or preparation for health talks remained invisible. This explains why use patterns appear inconsistent with the qualitative accounts of continued relevance. Therefore, declining log-in counts may not signal abandonment but rather a misalignment between what the metrics capture and what frontline workers actually value on the ground.

#### Theme 2: Acute Shocks and Everyday Constraints to Adoption

There were several barriers to the adoption of UpToDate, including acute shocks from calamities and fragile infrastructure and everyday constraints such as limited time, heavy workload, and literacy barriers.

##### Fragility Amid Calamities and Infrastructure Gaps (Wider System)

Even though UpToDate was designed with flexible features (offline access, mobile compatibility, and cross-cadre accounts), its adoption remained deeply vulnerable to wider systemic shocks. Participants from the remote site of Bulusan repeatedly emphasized how typhoons and fragile infrastructure disrupted sustained use. One physician explained the following:

We frequently face typhoons, impacting electricity and internet [access]. UpToDate is useful when accessible, but connectivity is challenging.

D01

Even when offline features were available, their utility was constrained by device limitations and repeated outages. A CHW explained that they had “two desktops, but they were damaged due to intermittent power outages” (C19).

In contrast, rural Samal, though also hit by outages, showed faster recovery. A program coordinator explained the difference:

The success in the connected area of Samal was so high.... UpToDate [usage] was greater, telemedicine was also greater in Samal, mainly because they had connectivity. Bulusan was very difficult to connect.

P01

This divergence reflects not only baseline differences (the rural site had cable infrastructure, whereas the remote site relied on patchy mobile signal) but also relative resilience capacities across sites.

##### Everyday Constraints (Adopter and Organization)

Beyond the acute disruptions caused by typhoons and outages, participants described a set of everyday constraints that limited how UpToDate could be integrated into practice.

Time and workload pressures were the most immediate barrier. One physician observed that “when the work demand is high, [CHWs] tend to many patients and can only use UpToDate during free hours” (D01).

Language and literacy barriers compounded these pressures. UpToDate content was in English, and while some workers relied on Google Translate, this slowed down their ability to use information directly with patients. One CHW noted the following:

It would be better if it were in Tagalog. You could explain it [to patients] much more clearly.

C04

Local workarounds also emerged. Legal and ethical constraints were less salient at the local level. In practice, sharing log-ins was tolerated, even normalized, as a pragmatic response to limited access. One CHW remarked the following:

I just use other people’s devices to access [UpToDate].

C11

Another CHW explained how UpToDate supported not only patient education but also the sharing of credible information among CHWs themselves:

I appreciate UpToDate even if I don’t know how to use a phone. I can ask other colleagues about the topics they’ve read. UpToDate helps us pass on credible information to colleagues and patients.

C40

These communal practices demonstrate that adoption was not strictly individual but collective as workers adapted access to fit resource and skill constraints. In this sense, the tool was not simply used “as designed” but reshaped to fit local realities.

##### Quantitative Integration (Adoption Over Time)

The site-level comparison illustrates how systemic constraints left distinct signatures in use data. In the rural setting, interruptions appeared as short-lived dips in activity, often recovering within weeks. In the remote setting, interruptions cascaded into prolonged troughs, with some months registering near-zero activity. These patterns suggest that remoteness not only lowered baseline adoption but also eroded resilience against shocks. At the same time, account sharing also helps explain why log-in counts understate actual use as activities carried out collectively often left no trace in the data.

## Discussion

### Principal Findings

This mixed methods evaluation shows that UpToDate’s usefulness in primary care for CHWs goes beyond its marketed role as a clinician-facing decision support tool. Across sites, CHWs and program implementers reinterpreted the tool, using it as a capacity-building resource and source of credibility. However, use trajectories were strongly shaped by acute environmental shocks (eg, typhoons and outages), and communal practices were conditioned by resource constraints.

### Theoretical Implications

Our findings highlight how adoption and nonadoption were shaped by the interactions between the adopter and technology domains of the NASSS framework. Misalignment between CHWs’ formal roles and the intended use of the tool opened space for domestication. Domestication refers to how users appropriate technologies into everyday practice that reshape routines and the meaning of the tool over time [31]. In our case, while adoption indicators suggested low or declining use, domestication was evident as CHWs redefined UpToDate in 2 ways.

First, UpToDate functioned as a form of symbolic capital, an unintended consequence of CHWs’ precarious professional positioning. Symbolic capital, in this sense, arose when the use of digital tools themselves operated as a form of capital that signaled competence and supported status attainment [32]. The association with an internationally branded resource further amplified that symbolic capital as part of its value derived from its Western provenance and the global authority it carried [33].

Second, the tool was reworked into a communal rather than individual resource. Shared log-ins and shared devices (practices that might be framed as misuse in other contexts) emerged as pragmatic, equitable adaptations to scarcity. In other low- and middle-income countries, studies similarly note reliance on borrowed or shared phones to access mobile health services, particularly for sexual and reproductive health, even when privacy risks are significant [34]. In our context, where the tool carried no patient data, communal access reduced exclusion without raising clinical confidentiality concerns. In other clinical settings, in contrast, privacy and security considerations may take precedence.

These dynamics underscore a broader contrast in implementation strategies. In many high-income settings, digital health implementation often emphasizes co-design with end users before deployment [35]. In our lower-resource setting, co-design was constrained by structural and resource barriers: the cost and expertise to maintain a continuously updated evidence base and the weak value proposition where CHWs were not the primary intended users. Importantly, adaptation after implementation is not a failure mode as even co-designed interventions are routinely modified during real-world implementation to maintain fit and effectiveness [36]. In such contexts, domestication can substitute idealized co-design, making adaptation after deployment a significant pathway to alignment.

Finally, within the wider system and organization domains, our findings highlight environmental and institutional drivers of domestication. The built environment (ie, mountainous terrain, island geography, and increasingly frequent severe typhoons) directly constrained digital access, making infrastructural fragility a central barrier to adoption. Organizationally, embedding the DHI in group routines (eg, journal clubs and peer discussions) aligned with evidence that structured training and peer practices help CHWs appropriate and circulate health education content [37]. At the same time, these organizational activities also legitimized communal use of UpToDate. While high-income setting debates on the wider system often foreground legal and regulatory hurdles regarding advanced technologies [27,38], in our setting, infrastructure and climate exposure were the more immediate constraints.

### Practice and Policy Implications

MAU metrics are more informative than crude log-in counts as frequent log-ins may be important for clinical decision support but CHWs often use the tool for education or peer learning, where even monthly access can be meaningful. However, consistent with extant use studies, MAU remains an insufficient proxy for value [39]. Milne-Ives et al [39] have recommended multidimensional evaluation strategies for DHIs. For UpToDate, this would mean assessing not only how often CHWs log in but also whether they draw on the tool to prepare health talks or improve the quality of counseling. Such dimensions of value could be captured through periodic group discussions or short surveys with CHWs to complement routine use data.

Positioning the tool to support extending primary care to underserved areas highlights its relevance for universal health care reforms in the Philippines. For scale-up, spread, and sustainability, policymakers and funders applying health technology assessment under universal health care reforms will need to reframe how they assess “value for money.” Narrow cost-effectiveness analyses risk undervaluing benefits that accrue through patient trust and professional development. For UpToDate, relevant outcomes may include improved patient education and workforce development and retention. Frameworks for complex interventions (eg, that by Skivington et al [40]) recommend capturing these multilevel outcomes and revisiting them iteratively as interventions adapt [40].

Digital health strategies in low- and middle-income countries should also require resilience planning as a funded component of procurement and implementation. Contracts and budgets should include technical safeguards tailored to climate vulnerabilities, such as backup power for typhoon-related outages and offline caching. At the same time, procurement should reflect how tools such as UpToDate are used in practice: not only across cadres but also in collective formats such as peer learning sessions. For the Department of Health, this means negotiating subscription arrangements that accommodate multicadre access and embedding peer learning structures such as journal clubs into program design.

### Limitations

Vendor logs lacked complete role metadata for CHWs and midwives, which limited finer-grained quantitative analyses of adoption by cadre. While this restricts cadre-specific analysis, CHWs represented the large majority of this group (7807/8768 person-months, 89.03% overall; 4481/4760, 94.13% in the remote site; 3326/4008, 82.98% in the rural site), suggesting that observed patterns are more likely to reflect CHW activity. Notably, the most striking finding was the apparent nonadoption among these groups. Consistent with the “science of attrition” by Eysenbach [41], nonuse itself warrants explanation. By linking longitudinal use data with qualitative insights, we were able to interpret these patterns as reflections of measurement misalignment and systemic constraints rather than simple user disinterest.

The qualitative component drew partly on secondary focus group data collected for previous evaluations, and this may have constrained the generation of new themes. However, the collection of additional primary qualitative data and analysis alongside quantitative data enabled a more comprehensive understanding and allowed for the inclusion of broader participant types. That said, male CHWs were largely absent from the qualitative data, reflecting their scarcity in the workforce. As a result, the findings primarily represent the perspectives of female CHWs, which is consistent with the gendered composition of the CHW workforce but may limit the transferability of these findings to their male counterparts. Transcripts were translated from Bikol and Filipino into English, and while multilingual teams ensured accuracy, some culturally embedded meanings may not have been fully conveyed. Researcher involvement with PPCS implementation also posed a risk of insider bias. To address this, we used reflexive practices and cross-perspective triangulation to check interpretations against the data.

Finally, this study was limited to 2 sites, which constrains generalizability. These sites represent common resource-limited contexts in the Philippines, making the findings potentially transferable to similar primary care settings where CHWs face comparable infrastructural and organizational constraints.

### Conclusions

UpToDate’s value lay in how CHWs adapted it to their everyday practice as a resource for professional development and as a marker of credibility. These adaptations show that apparent nonuse in use logs may still conceal meaningful engagement, underscoring the need for multidimensional evaluation frameworks that capture such complexities. At the same time, uptake was constrained by infrastructural fragility and recurrent climate shocks, highlighting that resilience planning, both technical and social, must be incorporated into digital health strategies from the start. For global digital health, these findings point to the importance of complementing engagement metrics with indicators of learning and system resilience when assessing the success of digital tools in primary care.

## Appendix

Multimedia Appendix 1 Checklist for Mixed Methods Research Manuscript Preparation and Review.

Multimedia Appendix 2 Standards for Reporting Qualitative Research checklist.

Multimedia Appendix 3 Topic guide for the focus group with program implementers.

## Abbreviations

CHW: community health worker
DHI: digital health intervention
MAU: monthly active user
NASSS: nonadoption, abandonment, scale-up, spread, and sustainability
PPCS: Philippine Primary Care Studies
TAM: technology acceptance model

## References

[1] Lehmann U, Sanders D (2007). Community health workers: what do we know about them? The state of the evidence on programmes, activities, costs and impact on health outcomes of using community health workers. World Health Organization.

[2] Mallari E, Lasco G, Sayman DJ, Amit AM, Balabanova D, McKee M, Mendoza J, Palileo-Villanueva L, Renedo A, Seguin M, Palafox B (2020). Connecting communities to primary care: a qualitative study on the roles, motivations and lived experiences of community health workers in the Philippines. BMC Health Serv Res.

[3] Raven J, Wurie H, Idriss A, Bah AJ, Baba A, Nallo G, Kollie KK, Dean L, Steege R, Martineau T, Theobald S (2020). How should community health workers in fragile contexts be supported: qualitative evidence from Sierra Leone, Liberia and Democratic Republic of Congo. Hum Resour Health.

[4] Heisler M, Choi H, Palmisano G, Mase R, Richardson C, Fagerlin A, Montori VM, Spencer M, An LC (2014). Comparison of community health worker-led diabetes medication decision-making support for low-income Latino and African American adults with diabetes using e-health tools versus print materials: a randomized, controlled trial. Ann Intern Med.

[5] Duffy S, Norton D, Kelly M, Chavez A, Tun R, Ramírez MN, Chen G, Wise P, Svenson J (2020). Using community health workers and a smartphone application to improve diabetes control in rural Guatemala. Glob Health Sci Pract.

[6] Damian AJ, Robinson S, Manzoor F, Lamb M, Rojas A, Porto A, Anderson D (2020). A mixed methods evaluation of the feasibility, acceptability, and impact of a pilot project ECHO for community health workers (CHWs). Pilot Feasibility Stud.

[7] Zaman SB, Silva ND, Goh TY, Evans RG, Singh R, Singh R, Singh A, Singh P, Thrift AG (2022). Design and development of a clinical decision support system for community health workers to support early detection and management of non-communicable disease. BMJ Innov.

[8] Bradley-Ridout G, Nekolaichuk E, Jamieson T, Jones C, Morson N, Chuang R, Springall E (2021). UpToDate versus DynaMed: a cross-sectional study comparing the speed and accuracy of two point-of-care information tools. J Med Libr Assoc.

[9] Scaffidi MA, Khan R, Wang C, Keren D, Tsui C, Garg A, Brar S, Valoo K, Bonert M, de Wolff JF, Heilman J, Grover SC (2017). Comparison of the impact of Wikipedia, UpToDate, and a digital textbook on short-term knowledge acquisition among medical students: randomized controlled trial of three web-based resources. JMIR Med Educ.

[10] Banzi R, Liberati A, Moschetti I, Tagliabue L, Moja L (2010). A review of online evidence-based practice point-of-care information summary providers. J Med Internet Res.

[11] Kwag KH, González-Lorenzo M, Banzi R, Bonovas S, Moja L (2016). Providing doctors with high-quality information: an updated evaluation of web-based point-of-care information summaries. J Med Internet Res.

[12] Venkatesh V, Morris MG, Davis GB, Davis FD (2003). User acceptance of information technology: toward a unified view. MIS Q.

[13] Calderon Y, Sandigan G, Tan-Lim CS, De Mesa RY, Fabian NM, Rey MP, Sanchez JT, Dans LF, Galingana CL, Bernal-Sundiang N, Casile RU, Aquino MR, Poblete KE, Lopez JF, Zabala H, Dans AL (2024). Feasibility, acceptability and impact of a clinical decision support tool among primary care providers in an urban, rural and remote site in the Philippines. BMJ Open Qual.

[14] Campbell JI, Aturinda I, Mwesigwa E, Burns B, Santorino D, Haberer JE, Bangsberg DR, Holden RJ, Ware NC, Siedner MJ (2017). The technology acceptance model for resource-limited settings (TAM-RLS): a novel framework for mobile health interventions targeted to low-literacy end-users in resource-limited settings. AIDS Behav.

[15] Shachak A, Kuziemsky C, Petersen C (2019). Beyond TAM and UTAUT: future directions for HIT implementation research. J Biomed Inform.

[16] Greenhalgh T, Papoutsi C (2019). Spreading and scaling up innovation and improvement. BMJ.

[17] Greenhalgh T, Wherton J, Papoutsi C, Lynch J, Hughes G, A'Court C, Hinder S, Fahy N, Procter R, Shaw S (2017). Beyond adoption: a new framework for theorizing and evaluating nonadoption, abandonment, and challenges to the scale-up, spread, and sustainability of health and care technologies. J Med Internet Res.

[18] Lee SY, Iott B, Banaszak-Holl J, Shih SF, Raj M, Johnson KE, Kiessling K, Moore-Petinak N (2022). Application of mixed methods in health services management research: a systematic review. Med Care Res Rev.

[19] O'Brien BC, Harris IB, Beckman TJ, Reed DA, Cook DA (2014). Standards for reporting qualitative research: a synthesis of recommendations. Acad Med.

[20] (2021). 2020 Census of population and housing (2020 CPH) population counts declared official by the President. Philippine Statistics Authority.

[21] (2006). The world health report 2006: working together for health. World Health Organization.

[22] Braun V, Clarke V (2022). Toward good practice in thematic analysis: avoiding common problems and be(com)ing a knowing researcher. Int J Transgend Health.

[23] De Mesa RY, Hendrickson ZM, Tan-Lim CS, Elepaño A, Fabian NM, Lopez JF, Latkin CA, Dans LF, Rey MP, Dans AM (2025). Resilience, ingenuity, and identity: a multi-level analysis of the Filipino community health worker experience in rural and remote municipalities in the Philippines. PLOS Glob Public Health.

[24] Pokhrel P, Karmacharya R, Taylor Salisbury T, Carswell K, Kohrt BA, Jordans MJ, Lempp H, Thornicroft G, Luitel NP (2021). Perception of healthcare workers on mobile app-based clinical guideline for the detection and treatment of mental health problems in primary care: a qualitative study in Nepal. BMC Med Inform Decis Mak.

[25] Braun V, Clarke V (2008). Using thematic analysis in psychology. Qual Res Psychol.

[26] Greenhalgh T, Maylor H, Shaw S, Wherton J, Papoutsi C, Betton V, Nelissen N, Gremyr A, Rushforth A, Koshkouei M, Taylor J (2020). The NASSS-CAT tools for understanding, guiding, monitoring, and researching technology implementation projects in health and social care: protocol for an evaluation study in real-world settings. JMIR Res Protoc.

[27] Abimbola S, Patel B, Peiris D, Patel A, Harris M, Usherwood T, Greenhalgh T (2019). The NASSS framework for ex post theorisation of technology-supported change in healthcare: worked example of the TORPEDO programme. BMC Med.

[28] Galingana CL, De Mesa RY, Marfori JR, Paterno RP, Rey MP, Co EE, Celeste JT, Dans LF, Dans AM (2020). Setting core competencies of health workers towards quality primary care: proceedings of a national consultative workshop. Acta Med Philipp.

[29] De Mesa RY, Marfori JR, Fabian NM, Camiling-Alfonso R, Javelosa MA, Bernal-Sundiang N, Dans LF, Calderon YT, Celeste JA, Sanchez JT, Rey MP, Galingana CL, Paterno RP, Catabui JT, Lopez JF, Aquino MR, Dans AM (2023). Experiences from the Philippine grassroots: impact of strengthening primary care systems on health worker satisfaction and intention to stay. BMC Health Serv Res.

[30] Bernal-Sundiang N, De Mesa RY, Marfori JR, Fabian NM, Calderon YT, Dans LF, Rey MP, Sanchez JT, Galingana CL, Catabui JT, Paterno RP, Co EE, Dans AM (2023). Governance in primary care systems: experiences and lessons from urban, rural, and remote settings in the Philippines. Acta Med Philipp.

[31] Silverstone R, Haddon L, Silverstone R, Mansell R (1996). Design and the domestication of information and communication technologies: technical change and everyday life. Communication By Design: The Politics of Information and Communication Technologies.

[32] Rodríguez-Camacho JA, Linder M, Jütte D, Hennig-Thurau T (2024). Digital capital: importance for social status in contemporary society and antecedents of its accumulation. Comput Hum Behav.

[33] Basaran T, Olsson C (2017). Becoming international: on symbolic capital, conversion and privilege. Millenn J Int Stud.

[34] Laar AS, Harris ML, Khan MN, Loxton D (2024). Views and experiences of young people on using mHealth platforms for sexual and reproductive health services in rural low-and middle-income countries: a qualitative systematic review. PLOS Digit Health.

[35] Sanz M, Acha B, García MF (2021). Co-design for people-centred care digital solutions: a literature review. Int J Integr Care.

[36] Kirk JW, Nilsen P, Andersen O, Stefánsdóttir NT, Grønfeldt B, Brødsgaard R, Pedersen BS, Bandholm T, Tjørnhøj-Thomsen T, Pedersen MM (2021). Adaptations and modifications to a co-designed intervention and its clinical implementation: a qualitative study in Denmark. BMC Health Serv Res.

[37] Greuel M, Sy F, Bärnighausen T, Adam M, Vandormael A, Gates J, Harling G (2023). Community health worker use of smart devices for health promotion: scoping review. JMIR Mhealth Uhealth.

[38] Winter PD, Chico TJ (2023). Using the non-adoption, abandonment, scale-up, spread, and sustainability (NASSS) framework to identify barriers and facilitators for the implementation of digital twins in cardiovascular medicine. Sensors (Basel).

[39] Milne-Ives M, Homer S, Andrade J, Meinert E (2024). The conceptualisation and measurement of engagement in digital health. Internet Interv.

[40] Skivington K, Matthews L, Simpson SA, Craig P, Baird J, Blazeby JM, Boyd KA, Craig N, French DP, McIntosh E, Petticrew M, Rycroft-Malone J, White M, Moore L (2021). A new framework for developing and evaluating complex interventions: update of Medical Research Council guidance. BMJ.

[41] Eysenbach G (2005). The law of attrition. J Med Internet Res.

