# Supplementary material for: Adoption of an Electronic Decision Support Tool for Capacity Building of Community Health Workers: Mixed Methods Study
Source: JMIR Form Res. 2026 Jan 7;10:e69874. doi: 10.2196/69874 (PMC12824572; doi:10.2196/69874)
Supplement: Multimedia Appendix 1 [file formative_v10i1e69874_app1.docx]

## Multimedia Appendix 1

Checklist for Mixed Methods Research Manuscript Preparation and Review

| **Item** | **Page** |
| --- | --- |
| **Rationale and Description of MMR Design** |  |
| Provide a clear statement of the study purpose | 3 |
| Explicitly describe the MMR design in accordance with Creswell’s (2015) typology and use a diagram to illustrate the relationship and sequence of qualitative and quantitative research components | 4 |
| Justify why the MMR design is appropriate for meeting the study purpose | 4 |
| **Transparency in Describing Method Details** |  |
| Describe the study population(s) and sample(s); e.g., who, what, how many | 4-5 |
| Describe the sampling procedures (including inclusion and exclusion criteria, recruitment) | 4-5 |
| Describe qualitative data collection processes (how often data were collected, who collected the data, what kind of data collection instruments were used, how data were recorded—e.g., notes, transcripts) | 4-6 |
| Describe quantitative data collection processes (how often data were collected, who collected the data, what kind of data collection instruments were used measurements, validity/reliability) | 4-6 |
| Describe qualitative data analysis processes (coding, single or multiple coders, replication logic, credibility) | 5-6 |
| Describe quantitative data analysis procedures (missing data and how they are handled, statistical tests used) | 4-5 |
| **Integration of Qualitative and Quantitative Research Components** |  |
| Interpret qualitative analysis results with appropriate quotes if necessary | 8-14 |
| Interpret quantitative analysis results in consideration of statistical significance, selection bias, and threats to validity | 6-7 |
| Compare qualitative and quantitative results | 8-14 |
| Address divergencies and inconsistencies between qualitative and quantitative results | 8-14 |
