# Supplementary material for: Adoption of an Electronic Decision Support Tool for Capacity Building of Community Health Workers: Mixed Methods Study
Source: JMIR Form Res. 2026 Jan 7;10:e69874. doi: 10.2196/69874 (PMC12824572; doi:10.2196/69874)
Supplement: Multimedia Appendix 3 [file formative_v10i1e69874_app3.docx]

## Multimedia Appendix 3

Topic Guide for Focus Group with Program Implementers

1. Condition
   1. For which health conditions do you think UpToDate is most helpful when used by community health workers (CHWs)?
   2. Do patients with these conditions have social or cultural factors that need to be taken into account when CHWs use UpToDate?
   3. How do you think these conditions, and the populations they affect, may change over the next 3–5 years?
2. Technology
   1. Can you describe the different use cases for UpToDate?
   2. Are there alternative providers of similar services or products?
   3. How would you rate UpToDate’s applicability in the Philippine primary care setting?
   4. Are there key technical interdependencies? Probes: integration with electronic health records, upgrading IT systems, satellite internet expansion (EO 127), journal clubs.
3. Adopter System
   1. To what extent do you think the technology and service model may become obsolete or require updating in the next 3–5 years?
   2. How do you think UpToDate affects the social status of CHWs in their communities? Probes: toward patients, colleagues and other healthcare providers, local government officials.
   3. How do you anticipate individual users’ perceptions of the technology will change over the next 3–5 years?
4. Value Proposition
   1. What is the value of the technology for patients? Probes: benefits, harms.
   2. What is the value of the technology for CHWs? Probes: does it create additional work, do benefits outweigh the inconvenience?
   3. What is the value of the technology for the Department of Health (DOH)? How might this technology be funded?
   4. What is the value proposition for local government units (LGUs) to support its use?
   5. What would be the business case for Wolters Kluwer to adapt UpToDate for the Philippine primary care setting? Probes: offering a Filipino-language interface, restricting access to patient information sections.
   6. Could the technology generate negative value (i.e., costs greater than benefits) for some stakeholders?
   7. Is the value proposition likely to change over the next 3–5 years?
5. Organization
   1. How would you rate the national and local governments’ capacities to adopt technological innovations such as the PPCS program on UpToDate?
   2. To what extent would organizational routines, pathways, or processes need to change to accommodate the technology?
   3. To what extent might DOH or LGUs undergo significant restructuring or leadership changes in the next 3–5 years that could impact the program?
6. Wider System
   1. What do you think of the political and policy climate as it relates to this innovation (e.g., UpToDate as part of the Expanded National Practice Guidelines Program, AO No. 2023-0002)?
   2. Do you think DOH is likely to support or oppose this innovation?
   3. Do you think LGUs are likely to support or oppose this innovation?
   4. Is the regulatory context supportive or adverse for this innovation?
   5. How might environmental factors, such as natural disasters and community resilience, affect adoption of the technology?
   6. How do you think the environmental, policy, regulatory, and economic context for this innovation may change over the next 3–5 years? Is turbulence likely?
